# Development of an Innovative Nutraceutical Fermented Beverage from Herbal Mate (*Ilex paraguariensis* A.St.-Hil.) Extract

**DOI:** 10.3390/ijms13010788

**Published:** 2012-01-13

**Authors:** Isabela Ferrari Pereira Lima, Juliano De Dea Lindner, Vanete Thomaz Soccol, José Luiz Parada, Carlos Ricardo Soccol

**Affiliations:** 1 Bioprocess Engineering and Biotechnology Division, Chemical Engineering Department, Federal University of Paraná, Curitiba, PR 81531-991, Brazil; E-Mails: isa.m.ferrari@gmail.com (I.F.P.L.); juliano@incorporefoods.com.br (J.D.D.L.); 2 Research and Development Department, Incorpore Foods, Camboriú, SC 88340-000, Brazil; 3 Post Graduation Program, Positivo University, Curitiba, PR 81280-330, Brazil; E-Mails: vasoccol@ufpr.br (V.T.S.); parada@up.com.br (J.L.P.)

**Keywords:** probiotic beverage, herbal mate, caffeine, antioxidant activity, *Lactobacillus acidophilus*

## Abstract

Herbal mate (*Ilex paraguariensis* A.St.-Hil.) leaves are traditionally used for their stimulant, antioxidant, antimicrobial, and diuretic activity, presenting as principal components polyphenolic compounds. The aim of this work was to develop an innovative, non-dairy, functional, probiotic, fermented beverage using herbal mate extract as a natural ingredient which would also be hypocholesterolemic and hepatoprotective. Among different strains used, *Lactobacillus acidophilus* was selected as the best for fermentation. The addition of honey positively affected the development of *L. acidophilus* and the formulated beverage maintained microbial stability during shelf life. Key ingredients in the extract included xanthines, polyphenols and other antioxidants with potential health benefits for the consumer. Caffeine levels and antioxidant activity were also studied. Acceptable levels of caffeine and large antioxidant capacity were observed for the formulation when compared to other antioxidant beverages. An advantage of this product is the compliance to organic claims, while providing caffeine, other phyto-stimulants and antioxidant compounds without the addition of synthetic components or preservatives in the formulation. Sensorial analysis demonstrated that the beverage had good consumer acceptance in comparison to two other similar commercial beverages. Therefore, this beverage could be used as a new, non-dairy vehicle for probiotic consumption, especially by vegetarians and lactose intolerant consumers. It is expected that such a product will have good market potential in an era of functional foods.

## 1. Introduction

The herbal mate (*Ilex paraguariensis* A.St.-Hil.) is a tree of the Aquifoliaceae family naturally distributed in South America. Aqueous extract of *Ilex paraguariensis* is a typical antioxidant-containing beverage largely consumed in several South American countries. In the USA, herbal mate is listed as GRAS (generally recognized as safe) [1].

Herbal mate extract (HME) has received attention for its health benefits. Some studies have reported that herbal mate is hypocholesterolemic and hepatoprotective [2], a central nervous system stimulant, a diuretic and an antioxidant [1,3–7]. Other studies have suggested that numerous active phytochemicals identified may be responsible for its health benefits as well. The highest concentrations of bioactive compounds are polyphenols (chlorogenic acid) and xanthines (caffeine, theobromine and theophylline) [8], which are responsible for antioxidant [1,9] and central nervous system stimulant effects [6].

Because most strains of lactic acid bacteria (LAB) are considered generally-recognized-as-safe (GRAS), there has been a long history for their widespread use in fermented foods [10]. Members of the genus *Lactobacillus* have been shown to colonize the human gastrointestinal tract. Several species are considered to exhibit beneficial effects, such as antimicrobial activity by acidification and by the production of bacteriocins, which inhibit the growth of food deteriorating and poisoning bacteria [11], anti-carcinogenic activity and their ability to enhance the immune response [12]. Furthermore, Kaizu *et al.* [13] demonstrated that some *Lactobacillus* species possessed antioxidant activity and were able to reduce the accumulation of reactive oxygen species (ROS) during the ingestion of food.

Traditionally, the products proposed for gastrointestinal health have been more popular in Europe (current estimated market at USD 8 billion), Japan and USA, than Latin America, Africa and Asia. Latin American consumers have recently begun to increase the consumption of functional prebiotic and probiotic foods and beverages that, when consumed regularly, could lead to better gastrointestinal health. For example, in 2008, the Brazilian probiotic beverage market was USD 250 million, with an increase of 11%, compared to 2007, which corresponded to 0.6% of the entire food market [14,15]. The Brazilian Association of the Food Industry (ABIA) predicted an increase of 13% for 2010 in the functional foods market, compared to a 6% increase for the entire food market. Brazil is the eighth major market in the world for this type of beverage [15].

Nowadays, there are an increasing number of herbal mate products being developed. The main objective of this study was to develop a functional, innovative, non-dairy probiotic, fermented beverage using HME as a natural ingredient. HME and carbohydrate substrates were fermented by different strains of probiotic LAB, and the final formulation was tested for the presence of bioactive compounds during the extended shelf life. Sensorial comparisons with other commercial beverages containing herbal mate were also examined.

## 2. Results and Discussion

### 2.1. Selection of the Formulation and LAB Strain

The first aspect of this study was to test and to select the best formulation of the herbal mate fermented probiotic beverage. Preliminary tests included changing HME concentrations and decoction conditions (data not shown). Using sensorial and physical criteria, 14 formulations were screened (Table 1) after fermentation using *Lactobacillus acidophilus*, *Lactobacillus sake*, *Lactobacillus casei* subsp. *rhamnosus* and *Lactobacillus casei*. After the analysis of appearance, bacterial development, precipitate formation, taste and odor, unacceptable formulations were eliminated and selected the formulation N (Table 1) fermented by *L. acidophilus*, which contained only HME and honey as the nitrogen and carbohydrate source. This formulation resulted in a sweet taste, moderate acidity, without bottom precipitate and was, thus, demonstrated to be a good substrate for the development of the probiotic strain *L. acidophilus* ATCC 4356.

### 2.2. Probiotic Fermentation of the HME

Among LAB, *L. acidophilus* has attracted attention for its potential probiotic effects in human health [16–18] and ability to use prebiotic compounds [19]. When selecting a probiotic culture for use as a dietary adjunct in human food, a number of factors should be considered. One of these is the use of a microorganism originating from the human intestinal tract that exhibits host specificity [20]. Additional factors important in selecting a strain that can survive and grow in the intestinal tract include bile and temperature tolerance, adherence capability to the intestinal wall, cholesterol assimilation and antioxidant ability [18,21,22].

**Figure 1:**
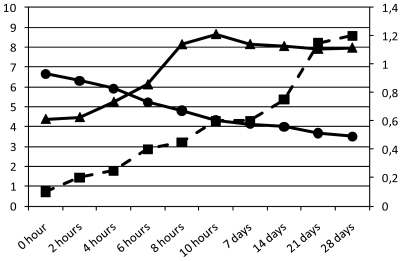
Changes in pH (●), growth trends of *Lactobacillus acidophilus* ATCC 4356 expressed as log colony-forming units (CFU) mL^−1^(▴) and acidity expressed as mL of 0.1 N NaOH (secondary axis—dashed line ■) during different steps of fermentation and shelf life for the herbal mate beverage.

*L. acidophilus* ATTC 4356, a human isolate, is used as a dietary adjunct in various cultured dairy products. In the present work, strain ATCC 4356 was used to ferment a non-dairy formulation, and showed to not only meet the strict criteria of viability but also capable of surviving storage. *L. acidophilus* cultivability was evaluated during fermentation and following shelf-life. Figure 1 showed the growth of *L. acidophilus*, where it was demonstrated to grow exponentially during 10 h of fermentation. During the shelf life period, the microbial count remained similar and was estimated at 10^8^ colony-forming units (CFU) mL^−1^. The presence of sufficient numbers of viable bacterial cells (10^9^ CFU) is necessary to provide therapeutic benefits [23]. Therefore, in order to call a product “probiotic”, such as in a new beverage, the viability of probiotic bacteria must be maintained. The formulated beverage maintained microbial stability during the 28 days of shelf life at 4 °C. The stable logarithmic probiotic value of 10^8^ CFU ml^−1^ found during the shelf life may prevent the development of deteriorative/poisoning microorganisms and deliver a sufficient concentration of probiotics to the consumer.

**Figure 2:**
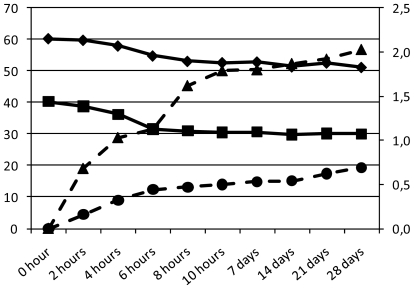
Sugar content (Glucose: solid line ■; Fructose: solid line ◆) and organic acids (secondary axis: Lactic acid—dashed line ▴; Acetic acid—dashed line ●) expressed as g L^−1^ for formulations and beverage during fermentation and shelf life.

*L. acidophilus* was shown to easily ferment a substrate rich in glucose and fructose (honey) and to produce lactic and acetic acid (Figure 2) resistant to acidic conditions after fermentation. The successful use of *L. acidophilus* resulted in a complete fermentation process. The optimum fermentation period of 10 h was determined after longer fermentation tests (data not shown). pH and bacterial growth analyses after 12 h showed a substantial decrease of viability of the cells in an acidic condition at pH 3.67.

Virtanen *et al.* [16] and Lin and Chang [24] demonstrated that *L. acidophilus* ATCC 4356 had the ability to scavenge DPPH free radicals. In the present study, antioxidant activities measured directly in the beverage varied from 51 to 62% (Table 2). No positive trend was observed for the confirmation of summation activity from bacterial or lysed cells in the HME.

### 2.3. Caffeine Content

HPLC analysis for caffeine content in the formulation for 10 h and in the beverage during shelf life indicated almost the same quantitative composition. The average values for each sample are presented in Table 2, based on chromatography using a caffeine standard.

The concentration of caffeine in relation to consumer consumption has been found to be approximately 6.7 mg in one regular dose (100 mL) of beverage (Table 2). Comparing coffee and herbal mate tea (approximately 56 and 52 mg per dose, respectively) [25] with this beverage, 6.7 mg of caffeine was detected, a low amount for ingestion of this alkaloid. Caffeine is one of the most studied alkaloids in terms of physiological effects in human beings [26,27]. Some physiological effects associated with caffeine include central nervous system stimulation, acute elevation of blood pressure, increased metabolic rate, and diuresis [28]. The values observed in the present product indicated that consumption of it provided a little intake of caffeine, an amount so small that not affect consumer preference.

### 2.4. Antioxidant Activity

The consumption of herbal mate significantly contributed to the overall antioxidant intake with potentially beneficial biological effects for human health [5]. A number of therapeutic applications have been claimed for herbal mate infusions. *I. paraguariensis* was shown to contain high antioxidant activity which positively correlated with the concentration of caffeoyl-derivatives [1,3]. Newell *et al.* [29] demonstrated that herbal mate tea possessed a much higher antioxidant capacity than green tea. According to Bixby *et al.* [3], herbal mate tea showed greater inhibition against cytotoxicity compared to green tea and or red wine. The ability to quench reactive oxygen species (responsible for cell structure damage after environmental oxidative stress) was examined and correlated to peroxidase-like activity which was related to the polyphenol concentration in herbal mate extract [30]. Higher polyphenol concentrations demonstrated greater peroxidase-like activity [31].

Quenching of the DPPH free radical by the formulations used in this study is shown in Table 2. The formulation during fermentation and beverage during shelf life showed similar amounts of antioxidant activity, approximately 56%. Comparison of the beverage with the study conducted by Ramadan-Hassanien [32] found that the anti-radical performance towards DPPH radicals of the fermented herbal mate beverage was just below those of mango and red grape juice, both rich in phenolic compounds. In other antioxidant beverages, such as hot drinks, especially those rich in caffeine [32], the product demonstrated a large antioxidant capacity, slightly below that of tea with lemon, green tea, black tea, soluble coffee and Turkish coffee.

### 2.5. Sensorial Analysis of the Beverage

**Figure 3:**
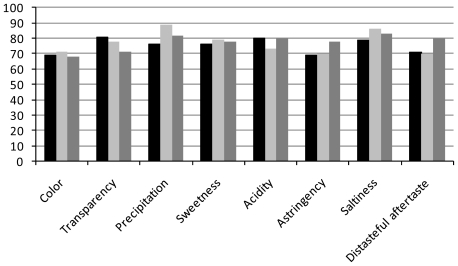
Acceptability factors in percentage (*Y* axis) and attributes (*X* axis) for the herbal mate beverage (black bar) and commercial mate tea A (dark grey bar) and B (pale grey bar).

The acceptance level for the probiotic and herbal mate Tea beverages is presented in Table 3 and supported by variance analysis (Table 4). Results for the sensorial analysis are expressed as a mean value. The mean value of some attributes presented significant differences and could be observed in the frequency distribution for the values of acceptability factors shown in Figure 3. Sweetness, acidity and astringency attributes for all beverages showed a similar acceptance by the testers. Significant differences for transparency and precipitation were expected when comparing fermented to an unfermented beverage, and these differences did not affect the final product acceptance (AF 71 and 82%, respectively).

According to Dutcosky [33], AF ≥ 70% represented good acceptability for the attribute analyzed in a sensorial analysis (Figure 3), and excluding color that presented an AF of 68%, this beverage presented a minimum AF of 71%.

For an overall evaluation, the average values for the sensory analysis and acceptability factors were kept within the acceptance range. No emergent alteration in the values at the final storage period (28 days) was found when compared to the product after fermentation (data not shown). Therefore, product acceptance could be considered as good and comparable to that of commercial beverages available at the local market.

## 3. Experimental Section

### 3.1. Preparation of the HME

Roasted, milled and sifted leaves and stems of herbal mate (*Ilex paraguariensis* A.St.-Hil.) were obtained commercially from the local markets in Santa Catarina State (Brazil). HME was prepared by decoction of 15 g in 300 mL of water at 95 °C for 3 min. The extract was filtered under vacuum using a filter paper (Whatman, UK).

### 3.2. HME Formulation and LAB Strains

The formulations were composed by 300 mL of HME and one or two carbohydrate substrates in the proportions described in the Table 1. Honey (Mel de Abelha Superbom, São Paulo, Brazil), malt extract (Acumedia, USA), yeast extract (Acumedia, USA) and sugar cane molasses (local bioethanol producing company) were used with the aim to prepare formulations for the LAB fermentation.

*L. acidophilus* ATCC 4356, *L. sake* ATCC 15521, *L. casei* ATCC 393 and *L. casei* subsp. *rhamnosus* DEBB H-19 (obtained from the Culture Bank of the Bioprocess Engineering and Biotechnology Division, Federal University of Paraná, Brazil) were used in this work. They were grown overnight in MRS (De Man, Rogosa and Sharpe) broth (Acumedia, USA) at 35 °C. The strains were inoculated at a standard concentration of 10^6^ colony-forming units (CFU) mL^−1^ in 300 mL (3%) of the formulations indicated in Table 1.

### 3.3. pH and Acidity

The pH was measured directly using a model HI9321 pH meter (Hanna Instruments, Portugal).

Acidity was measured by titrimetric analysis using the fermented broth diluted 1:50 (v/v) in distilled water. The diluted solution was neutralized with 0.1 M NaOH (Merck, Germany) using an alcoholic solution of phenolphthalein (1% v/v) as an indicator.

### 3.4. Sugars and Organic Acids Concentration

The fermented broths were diluted 1:10 in MilliQ^®^ water and filtered using a 0.22 μm pore filter (Millipore, UK). Sugars (glucose and fructose) and organic acids (lactic and acetic) content were determined by HPLC. Chromatographic analyses were performed using a LC-10AD chromatographic system (Shimadzu, Japan) equipped with a model RID-10A detector (Shimadzu, Japan) and an Aminex HPX-87H cation exchange column (300 mm × 7.8 mm i.d., Bio-Rad Laboratories, CA, USA). Analyses were performed using filtered, degassed 5 mM reagent grade H_2_SO_4_ (Carlo Erba, Italy) as the mobile phase at a flow rate of 0.6 mL·min^−1^. Eluates were monitored at 215 nm. Calibration curves were obtained by preparing a standard mix of the sugars and organic acids (Sigma, USA). The resulting peaks area were calculated for duplicate 25 μL injections and plotted against concentration. Each assay was carried out in duplicate and the average values expressed in g·L^−1^ for glucose, fructose, lactate and acetic acid during fermentation were calculated. The estimated error for the chromatographic assays was less than 3%.

### 3.5. LAB Count and Fermentation

Formulations were serially diluted tenfold in 0.05 mol·L^−1^ sodium citrate (Sigma, USA) buffer, pH 7.5. In order to quantify the cultivable LAB population, MRS agar (Acumedia, USA) was used and incubated at 35 °C for 48 h under anaerobic conditions. Bacterial counts were carried out in triplicate and the standard deviation of mean values was calculated. The estimated error was less than 10%.

After fermentation, the herbal mate probiotic beverage was stored in Scotch flasks at 4 °C during 28 days and the parameters as above were analyzed after 0, 7, 14, 21 and 28 days.

### 3.6. Caffeine Content

The caffeine content, present in the beverage during shelf life, was analyzed using an Agilent 1100 HPLC system (Hewlett Packard, Inc., USA) with a diode array detector (DAD). Chromatographic separation was accomplished using a Zorbax RX Reversed-Phase C18 column, 150 mm × 4.6 mm (Agilent, USA). An isocratic system (MeOH-H_2_O 30:70) was used as the mobile phase at a flow rate of 1.0 mL·min^−1^ at 35 °C. Detection was performed at 272 nm. Broth and beverage were both diluted (5 mL in 250 mL MilliQ water), filtered through 0.22 μm (Millipore, UK) and injected in triplicate (CV < 5%). Peak areas were compared to standard curves. Suitable amounts of caffeine standard were dissolved in MeOH-H_2_O (4:6). Standard solutions were injected in triplicate and peak areas were measured. Linearity was evaluated by linear regression analysis, and the precision and accuracy were determined by the coefficient of variation (CV < 4%). The correlation coefficient was *r* = 0.988.

### 3.7. Antioxidant Activity

The antioxidant activity (free radical scavenging capacity) present in the beverage, during the shelf life, was examined by the reduction of the 1,1-diphenyl-2-picrylhydrazyl radical (DPPH) as described by Ramadan *et al.* [34] with modifications. Five millilitres of a 20 mg·mL^−1^ DPPH solution in methanol were added to 5 mL of a methanolic solution of the fermented beverage (1:10, v/v). Absorbance was determined spectrophotometrically (UV-1601PC Spectrophotometer, Shimadzu, Japan) at 515 nm after 30 min., and scavenging activity was calculated as percent of radical reduction. The percent of inhibition was defined as [(A517 blank – A517 sample) A517 blank^−1^]·100^−1^ (%). Ascorbic acid was used as a reference compound. The analysis was carried out in triplicate and the standard deviation of mean values was calculated.

### 3.8. Sensorial Analysis

Sensorial evaluation of the beverage was performed on the product after 28 days of shelf-life. Sensorial characteristics of the beverage were compared with two commercial herbal mate lemon flavored teas. The commercial products were purchased from a local market. Mate teas A and B correspond to two different commercial brands.

Sensorial hedonic test of the beverage was carried out by a group of 15 non-trained testers who judged the color, transparency, precipitate formation, sweetness, acidity, astringency, saltiness and distasteful after taste using a hedonic rating scale from 1 to 9 (1: dislike extremely; 2: dislike very much; 3: dislike moderately; 4: dislike slightly; 5: neither like nor dislike; 6: like slightly; 7: like moderately; 8: like very much; 9: like extremely). Sensory tests were performed in individual booths, under white light during the morning shift (9:00 a.m.–11:30 a.m.). Refrigerated (5 °C) samples were served in transparent glass cups. The data obtained were analyzed by ANOVA and Tukey’s test according to Monteiro [35] using Assistat version 7.5 software (Assistat, Brazil).

To verify the acceptability of the tested beverages, an acceptability factor (AF) [33] was calculated as the criteria to evaluate each sensorial attribute analyzed:

$$\text{AF}=A\cdot 100\cdot B^{-1}$$

where *A* is the mean value obtained for each attribute; *B* is the maximum value ascribed by the testers for each attribute.

## 4. Conclusions

The interest in herbal mate has risen in recent years because it has shown extraordinary possibilities not only as a consumer beverage but also as a nutraceutical product in the novel food industry. The present work demonstrated a process for preparing a non-dairy, probiotic, fermented beverage from HME. The base formulation ingredients incorporated xanthines, polyphenols and other antioxidant components that have a potential for healthy consumer benefits. The innovative probiotic beverage contained sufficiently high viable counts of *L. acidophilus*. The addition of honey positively affected the development of *L. acidophilus*, and the formulated beverage maintained microbial stability during storage with a stable logarithmic probiotic value of CFU mL^−1^ during shelf life. Acceptable levels of caffeine and large antioxidant capacity were observed for the formulation when compared to other antioxidant beverages. An advantage of this product is the compliance to organic claims, while providing caffeine, other phyto-stimulants and antioxidant compounds without the addition of synthetic components or preservatives in the formulation. Sensorial analysis demonstrated that the beverage had good consumer acceptance in comparison to two other commercial beverages. This beverage can be used as a new, non-dairy vehicle for probiotic consumption, especially by vegetarians and lactose intolerant consumers. The beverage product could have an excellent market potential in the current era of new functional foods.

## Figures and Tables

**Table 1:** Formulations with different compositions of carbohydrate and nitrogen substrates.

Formulations	Carbohydrate and nitrogen substrates (w/v) a			

	Yeast extract	Honey	Malt extract	Sugar cane molasse
A	0.5	4	-	-
B	0.5	6	-	-
C	0.5	8	-	-
D	0.5	10	-	-
E	0.5	4	-	-
F	1.5	4	-	-
G	-	-	4	-
H	-	-	6	-
I	-	-	8	-
J	-	-	-	4
K	-	-	-	6
L	-	-	-	8
M	-	10	-	-
N	-	14	-	-

a percentage; - absent.

**Table 2:** pH and mean values obtained after duplicate assays for fermentation parameters, caffeine content and antioxidant activity of formulation during fermentation and beverage during shelf life.

	pH	Acidity a	Bacterial count b	Sugar c		Organic acid c		Caffeine d	Antioxidant activity e

				Glucose	Fructose	Lactic	Acetic		
0 h	6.65	0.10	4.37 ± 3.12	40.2	60.2	0.00	0.00	nd	nd
2 h	6.30	0.20	4.46 ± 2.15	38.7	59.7	0.68	0.16	nd	nd
4 h	5.92	0.25	5.23 ± 3.94	36.2	57.9	1.03	0.32	nd	nd
6 h	5.23	0.40	6.11 ± 4.18	31.5	54.8	1.13	0.44	nd	nd
8 h	4.80	0.45	8.13 ± 3.24	30.9	53.1	1.62	0.47	nd	nd
10 h	4.30	0.60	8.64 ± 5.14	30.5	52.7	1.79	0.50	6.56	58 ± 2
12 h	3.67	0.70	7.69 ± 4.47	30.1	52.6	1.94	0.53	nd	nd
7 days	4.12	0.60	8.14 ± 4.56	30.6	52.9	1.80	0.53	6.70	56 ± 3
14 days	4,00	0.75	8.04 ± 2.98	29.8	51.4	1.87	0.54	6.82	55 ± 2
21 days	3.67	1.15	7.90 ± 3.35	30.1	52.6	1.92	0.62	6.68	59 ± 2
28 days	3.50	1.20	7.96 ± 4.16	30.0	51.2	2.03	0.69	6.70	58 ± 1

a mL 0.1 N NaOH;

b logarithmic bacterial counts expressed as log CFU mL^−1^ (±SD);

c g L^−1^;

d mg 100 mL^−1^;

e % reduction DPPH (±SD), percentage inhibition defined as [(A517 blank − A517 sample) A517 blank^−1^] 100^−1^ (%); nd: not determined.

**Table 3:** Acceptance (average values ± SD) and acceptability factors (AF) for the herbal mate beverage and commercial mate tea A and B.

Attributes (AF)	Commercial Mate Tea A	Commercial Mate Tea B	Probiotic Mate Beverage
Color	6.2 ± 1.4 a (69)	6.4 ± 1.3 a (71)	5.4 ± 1.3 b (68)
Transparency	7.3 ± 1.3 a (81)	7.0 ± 1.2 a (78)	5.0 ± 1.2 b (71)
Precipitation	7.8 ± 1.1 a (76)	8.0 ± 1.1 a (89)	4.9 ± 1.3 b (82)
Sweetness	6.1 ± 1.4 a (76)	6.3 ± 1.3 a (79)	5.5 ± 1.4 a (78)
Acidity	6.4 ± 1.1 a (80)	6.6 ± 1.3 a (73)	5.6 ± 1.0 a (80)
Astringency	6.2 ± 1.7 a (69)	6.3 ± 1.7 a (70)	5.5 ± 1.3 a (78)
Saltiness	7.1 ± 1.1 a (79)	6.9 ± 1.0 ab (86)	5.8 ± 1.0 b (83)
Distasteful aftertaste	6.4 ± 1.3 a (71)	6.3 ± 1.5 b (70)	6.4 ± 1.2 a (80)

a b Means with the same letter in the same line are not significantly different (*P* < 0.05), according to ANOVA and Tukey’s test.

**Table 4:** Variance analysis (ANOVA) for the sensorial test of beverages.

Source of variation	Degree of freedom	Sum of squares	Mean of squares	*F* ratio
Color	2	5.6000	2.8000	1.7571 *
Transparency	2	31.2666	15.6333	9.8106 **
Precipitation	2	60.2000	30.1000	18.8890 **
Sweetness	2	3.4666	1.7333	1.0877 ns
Acidity	2	5.6000	2.8000	1.7571 ns
Astringency	2	3.8000	1.9000	1.1923 ns
Saltiness	2	9.8000	4.9000	3.0750 *
Distasteful aftertaste	2	0.0666	0.0333	0.0209 *
Residue	216	344.2000	1.5935	

Total	232	464.0000		

** 1% significance (*P* < 0.01);

* 5% significance (0.01 ≤ *P* < 0.05);

ns not significant (*P* ≥ 0.05).
